# Characterisation of the bifunctional dihydrofolate synthase–folylpolyglutamate synthase from *Plasmodium falciparum*; a potential novel target for antimalarial antifolate inhibition

**DOI:** 10.1016/j.molbiopara.2010.03.012

**Published:** 2010-07

**Authors:** Ping Wang, Qi Wang, Yonghong Yang, James K. Coward, Alexis Nzila, Paul F.G. Sims, John E. Hyde

**Affiliations:** a Manchester Interdisciplinary Biocentre, Faculty of Life Sciences, University of Manchester, 131 Princess Street, Manchester M1 7DN, UK; b Department of Medicinal Chemistry, University of Michigan, 930 N. University, Ann Arbor, MI 48109-1055, USA; c Department of Chemistry, University of Michigan, 930 N. University, Ann Arbor, MI 48109-1055, USA; d KEMRI, Wellcome Trust Collaborative Research Programme, Kilifi 80108, Kenya

**Keywords:** Antifolate inhibitor studies, Biphasic kinetics, Folate metabolism, Methotrexate, Phosphinate analogues of folate, Substrate specificity, DHF, dihydrofolate, DHFR, dihydrofolate reductase, DHFS, dihydrofolate synthase, DHP, dihydropteroate, DHPS, dihydropteroate synthase, DTT, dithiothreitol, FPGS, folylpolyglutamate synthase, GTPCH, GTP cyclohydrolase I, HPPK, hydroxymethyldihydropterin pyrophosphokinase, MTX, methotrexate, pAB, *para*-aminobenzoate, PTPS, pyruvoyltetrahydropterin synthase III, PYR, pyrimethamine, SDX, sulfadoxine, THF, tetrahydrofolate

## Abstract

Unusually for a eukaryote, the malaria parasite *Plasmodium falciparum* expresses dihydrofolate synthase (DHFS) and folylpolyglutamate synthase (FPGS) as a single bifunctional protein. The two activities contribute to the essential pathway of folate biosynthesis and modification. The DHFS activity of recombinant PfDHFS–FPGS exhibited non-standard kinetics at high co-substrate (glutamate and ATP) concentrations, being partially inhibited by increasing concentrations of its principal substrate, dihydropteroate (DHP). Binding of DHP to the catalytic and inhibitory sites exhibited dissociation constants of 0.50 μM and 1.25 μM, respectively. DHFS activity measured under lower co-substrate concentrations, where data fitted the Michaelis–Menten equation, yielded apparent *K*_m_ values of 0.88 μM for DHP, 22.8 μM for ATP and 5.97 μM for glutamate. Of the substrates tested in FPGS assays, only tetrahydrofolate (THF) was efficiently converted to polyglutamylated forms, exhibiting standard kinetics with an apparent *K*_m_ of 0.96 μM; dihydrofolate, folate and the folate analogue methotrexate (MTX) were negligibly processed, emphasising the importance of the oxidation state of the pterin moiety. Moreover, MTX inhibited neither DHFS nor FPGS, even at high concentrations. Conversely, two phosphinate analogues of 7,8-dihydrofolate that mimic tetrahedral intermediates formed during DHFS- and FPGS-catalysed glutamylation were powerfully inhibitory. The *K*_i_ value of an aryl phosphinate analogue against DHFS was 0.14 μM and for an alkyl phosphinate against FPGS 0.091 μM, with each inhibitor showing a high degree of specificity. This, combined with the absence of DHFS activity in humans, suggests PfDHFS–FPGS might represent a potential new drug target in the previously validated folate pathway of *P. falciparum*.

## Graphical abstract

The activities of the bifunctional folate pathway enzyme dihydrofolate synthase–folylpolyglutamate synthase from *Plasmodium falciparum* are characterised with respect to their kinetics, substrate specificities and responses to folate analogue inhibitors.

## 1 Introduction

The antifolates are clinically important drugs used to target both bacterial and eukaryotic pathogens, including the apicomplexan, *Plasmodium falciparum* (Pf), a parasite that still claims over a million lives each year and causes considerable economic loss to developing countries [1,2]. The antifolates that have been deployed against malaria target only two enzymes in the folate biosynthesis pathway of the parasite. Dihydropteroate synthetase (DHPS; EC 2.5.1.15), which catalyses the synthesis of 7,8-dihydropteroate (DHP) from 6-hydroxymethyl-7,8-dihydropterin pyrophosphate and *p*-aminobenzoate (pAB), is inhibited by sulfonamide and sulfone drugs, while dihydrofolate reductase (DHFR; EC 1.5.1.3), which reduces 7,8-dihydrofolate (DHF) to the active cofactor 5,6,7,8-tetrahydrofolate, is targeted principally by pyrimethamine (PYR) and cycloguanil. Inhibition of these activities, most commonly in a synergistic combination of sulfadoxine and PYR, disrupts the constant supply of tetrahydrofolate (THF) cofactors required for key 1-carbon transfer reactions, including synthesis of thymidylate, which is essential for DNA replication.

**Fig. 1:**
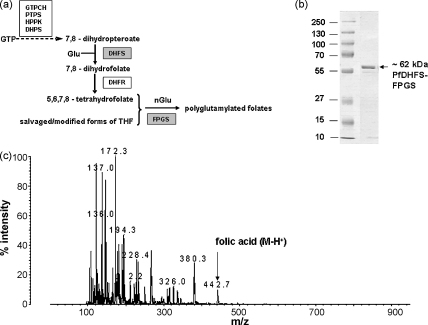
*P. falciparum* DHFS–FPGS: roles, expression of recombinant protein and product analysis. (a) Position (grey boxes) and roles of DHPS and FPGS activities in the folate pathway of *P. falciparum* leading to 5,6,7,8-tetrahydrofolate (THF). Polyglutamation of folates is thought to occur on their tetrahydro-forms. The addition of one or more glutamate residues by FPGS to THF or its modified forms, produced *de novo* or from salvaged host folates, is indicated by nGlu. The dotted arrow indicates sequential steps involving the four enzymes shown. (b) SDS-PAGE analysis of purified PfDHFS–FPGS after Ni-agarose affinity and ion-exchange chromatography; (c) mass spectral analysis of the reaction mix after a standard 1 h incubation of the DHFS assay.

The long-established efficacy of folate metabolism as a clinical target and the widespread resistance to current antifolates and other classes of antimalarials highlights the importance of identifying other targets within the *P. falciparum* folate/thymidylate biosynthesis pathways, which comprise a further seven enzyme activities in addition to DHPS and DHFR [3]. One activity of folate biosynthesis yet to be characterised in malaria parasites is dihydrofolate synthase (DHFS; EC 6.3.2.12), which adds an l-glutamate residue to the pAB component of DHP, the product of DHPS, to form DHF, the substrate of DHFR (Fig. 1a). DHFS represents a target unique to the parasite, as the human host is unable to synthesise folates and lacks this enzyme. Closely related to the activity of DHFS is that of folylpolyglutamate synthase (FPGS; EC 6.3.2.17), which adds further glutamate residues to reduced folate monoglutamates by γ-linkage (Fig. 1a), with the number of residues incorporated varying among organisms [4,5], ranging from an average of 3 in *Escherichia coli* [6] up to as many as 9 in *Lactobacillus casei* [7] and mammals [6]. In *P. falciparum*, the pentaglutamate form of 5-methyltetrahydrofolate has been identified as the predominant polyglutamylated folate metabolite [8,9], demonstrating the importance of FPGS catalysis in these organisms as well. Such conjugated forms are apparently ubiquitous and the crucial role of polyglutamylation has been demonstrated by studying organisms mutated in their *fpgs* genes. For example, CHO cells mutant in this gene require supplementation by the end-products of folate metabolism and exhibit much reduced levels of intracellular folates, predominantly as monoglutamates [10,11]. Similarly, the *MET7* gene encoding FPGS in *Saccharomyces cerevisiae* is essential for methionine biosynthesis and the maintenance of mitochondrial DNA [12]. In mammals and plants several folate-dependent enzymes exhibit much higher affinity for polyglutamylated folates compared to their monoglutamylated equivalents [4,13,14]. Another function of polyglutamylation is to prevent folates from leaking through the cell membranes and sub-cellular compartments by substantially increasing the negative charge they carry [10,15,16] and in human cells, polyglutamylation by FPGS has been shown to have a critical role in the cellular retention and enzyme targeting of the major anti-cancer drug and folate analogue methotrexate (MTX) [17,18].

Some bacteria, such as *Corynebacterium* species [19], *Neisseria gonorrhoeae* [20], *Mycobacterium tuberculosis* [21] and *E. coli* [22] express bifunctional DHFS–FPGS molecules that perform both the first and subsequent additions of glutamate. In *E. coli* and *M. tuberculosis*, recent work has shown that the active centres of the DHFS and FPGS functions co-localise closely, at least for addition of the first glutamate to THF by the latter, but are not identical [21,23,24]. However, in *L. casei* the FPGS has no accompanying DHFS activity [25] and folate must be salvaged. This is also the case in mammals, including humans [26,27], where pre-formed folate is an essential nutrient. In eukaryotes that can synthesise folate *de novo*, such as fungi and plants, both activities are present but are usually encoded by separate genes [28,29]. However, we have demonstrated previously that a single protein from *P. falciparum* carries both DHFS and FPGS activities, the first example of a bifunctional enzyme of this type from a eukaryotic organism [30].

The critical dual role of parasite DHFS–FPGS in both the biosynthesis and modification of folates, and the absence of DHFS activity in humans suggest the possibility that parasite-specific inhibitors targeted to this molecule might be feasible and effective. We therefore undertook a detailed study of PfDHFS–FPGS with respect to its kinetic properties, substrate specificities and susceptibility to the antifolate drug MTX, as well as to novel inhibitors based on phosphinic acid analogues of folic acid.

## 2 Materials and methods

### 2.1 Reagents

Specialty reagents were obtained commercially as follows: l-[U-^14^C] glutamic acid (238 mCi/mmol), ^3^H-folinic acid (30 Ci/mmol) and ^3^H-methotrexate (31.8 Ci/mmol) (Moravek Biochemicals, Inc., California); DE81 anion-exchange chromatography paper (Whatman International Ltd., UK); DHF, THF, folinic acid and DHP (Schircks Laboratories, Jona, Switzerland); 2-mercaptoethanol, sodium hydrosulfite (dithionite), folic acid, ATP, BSA, l-glutamic acid, and dithiothreitol (DTT) (Sigma), Overnight Express™ Instant TB Medium (Merck), Ni-NTA resin (Qiagen Ltd., UK). The *E. coli* expression host used was BL21(DE3) (Novagen). The isolates of *P. falciparum* used were K1, FCB, V1/s, Fcr3, as well as the cloned line 3D7. The aryl phosphinate folate analogue, 2-[[[4-[N-[(2-amino-3,4-dihydro-4-oxo-6-pteridinyl)methyl]amino]phenyl](hydroxy)phosphinoyl]methyl]pentane-1,5-dioic acid (compound **1**) [31] and the alkyl phosphinate folate analogue, 2-[[[3-[[4-[[(2-amino-3,4-dihydro-4-oxo-6-pteridinyl)methyl]amino]benzoyl]amino]-3-carboxypropyl]hydroxyphosphinyl]methyl]pentane-1,5-dioic acid (compound **2**) [32,33] were synthesised in the laboratory of JKC.

### 2.2 Expression and protein purification

A verified cDNA fragment encoding the entire *dhfs-fpgs* gene [30,34] was cloned from the K1 isolate of *P. falciparum* into pET22b (Novagen, UK) using the NdeI and BamHI sites and the construct transformed into *E. coli* BL21(DE3) host cells. Production of DHFS–FPGS was carried out by an autoinduction procedure [35] in Overnight Express instant medium (Novagen, UK). Incubation was at 37 °C overnight and then at 18 °C for a further 24 h. The cell pellet was resuspended in 50 mM sodium phosphate buffer, pH 8, with 300 mM NaCl at a concentration of up to 200 g wet weight/l, and processed with a high pressure cell breaker (Constant Cell Disruption System, Constant Systems Ltd., UK) at a pressure of 25,000 psi. Cell debris was removed by centrifugation and the supernatant collected. The His-tagged DHFS–FPGS was then purified on a Ni-NTA column and bound protein eluted with 250 mM imidazole. The protein was further purified on a Q-Sepharose high performance ion-exchange column, using a loading buffer of 25 mM Tris–HCl, pH 7.5 and eluting with a gradient from 0 to 1 M NaCl in the same buffer. The activity was monitored by the DHFS assay described below and the active peak area collected. Yields were ∼2 mg/l of starting *E. coli* culture. Glycerol (50%, v/v) was added to the protein, which was stored at concentrations of 0.5–1 mg/ml at −80 °C for future use.

### 2.3 DHFS and FPGS assays

A standard reaction mix based on those established for bacterial and eukaryotic DHFS/FPGS assays was adopted [28,36,37] and contained 10 mM MgCl_2_, 5 mM DTT, 200 mM KCl, 100 mM Tris, 50 mM glycine, 1 mg/ml BSA, 45 μg/ml DHFS–FPGS protein (0.72 μM), pH 10. For the three substrates of the reaction, up to 5 mM ATP, up to 1 mM l-[U-^14^C] glutamate (diluted from the commercial stock with unlabelled l-glutamate to the required specific activity) and up to 100 μM DHP (for DHPS) or THF (for FPGS) were used, depending upon the purpose of the assay, as indicated in the text. The enzyme reactions were carried out at 37 °C for 60 min in triplicate and stopped by transferring reaction tubes into an ice-cold water bath. Reaction mixes were then spotted onto DE81 ion-exchange paper (pre-treated with 0.5 M EDTA, pH 8.0 and then dried), air dried and the unreacted label washed away with 125 ml of 40 mM NaCl, 10 mM Tris–HCl, pH 8.0, four times, 20 min each wash. The filter was then exposed to a storage phosphor screen overnight and the screen scanned with a quantitative imager (Typhoon Trio Variable Mode Imager, GE Healthcare, UK). The measured counts were converted to concentrations by comparison with a standard curve derived from known amounts of the radiolabelled l-glutamate subsequently used as substrate in the assays. The enzyme activity data was analysed using the Matlab software package. Regression curves were fitted using the least squares routine.

### 2.4 Metabolic labelling of parasite cultures with ^3^H-folinic acid and ^3^H-methotrexate

0.5 μCi/ml of either ^3^H-folinic acid or ^3^H-methotrexate at the same specific activity was used to label parallel 50 ml synchronous cultures of *P. falciparum* FCB strain. Label was incubated with ring-stage (6–10 h) parasites, which were harvested at the late trophozoite stage 20 h later. The labelled products were extracted by the boiling method and analysed using HPLC on a C18 column eluted with an acetonitrile gradient as described [38].

### 2.5 MALDI-ToF mass spectrometry of DHFS–FPGS reaction products

The matrix used was α-cyano-4-hydroxycinnamic acid, which was dissolved in acetonitrile:water (1:1, v/v), 0.1% formic acid (v/v) at a concentration of 10 mg/ml. The reaction mix was partially purified with a C18 SPE device. One μl of the eluted material in 50% acetonitrile, 0.1% formic acid was mixed with the same volume of matrix solution and 1 μl of the mix placed onto the target and allowed to air dry. Spectra were acquired with an AXIMA-CFR MALDI-ToF spectrometer (Kratos Analytical Ltd., Manchester, UK), in positive reflectron mode with a power setting of 180. The spectra were obtained by overlapping 100 profiles. Mass to charge (*m*/*z*) calibration was performed externally using the matrix peaks and folyldiglutamate as standards.

### 2.6 Reduction of pteroyl phosphinates

Before evaluation as possible inhibitors, the pterin rings of compounds **1** and **2** (Section 2.1) were reduced with mild sodium hydrosulfite (dithionite) treatment to provide the corresponding 7,8-dihydro derivatives [39,40], as confirmed by MALDI-ToF spectrometry of the parent and reduced compounds (negative ion mode, **1** as [M−H]^−^ = 604.2, H_2_-**1** as [M−H]^−^ = 606.2; **2** as [M−H]^−^ = 474.4, H_2_-**2** as [M−H]^−^ = 476.4; negligible signals for H_4_X forms). For this reduction, the parent compounds were first dissolved in DMSO at 5 mM then reduced in a 50% aqueous solution containing sodium hydrosulfite (20 mg/ml) and 2-mercaptoethanol (10 mM). Mixes were kept at room temperature for 80 min, and then the compounds aliquoted, analysed and either used immediately or moved to −20 °C for short-term storage. A fresh aliquot was used for each assay.

## 3 Results

### 3.1 Expression of dihydrofolate synthase and folylpolyglutamate synthase

The *dhfs-fpgs* open reading frame obtained as cDNA from *P. falciparum* isolate K1 [34] was cloned, expressed and purified as described in Section 2. The purified His-tagged protein ran on SDS-PAGE close to its predicted molecular mass of 62.4 kDa (Fig. 1b). Identity and purity were confirmed by LC–MS–MS analysis of tryptic peptides from the purified protein (Mascot score = 468, against complete UNIPROT database; 31% sequence coverage, with no detectable peptides from any other protein, including *E. coli* DHFS–FPGS). Gel filtration experiments in non-denaturing conditions gave an apparent molecular mass of ∼90 kDa (data not shown), which although significantly higher than the calculated mass of a single chain, was too small to represent a dimer, indicating that the plasmodial protein is most likely to be monomeric, in common with all other DHFS/FPGS enzymes thus far reported from bacterial [22,25], fungal [28], plant [41] and mammalian [36,42] sources.

PfDHFS–FPGS represents a complex system where the closely related DHFS and FPGS activities each catalyse reactions in which there are three substrates, the pterin/folate moiety (DHP in the case of DHFS, THF mono- or polyglutamylated forms in the case of FPGS), ATP and glutamate. To establish initial conditions, the enzyme was tested separately for DHFS and FPGS activities using saturating concentrations of the natural substrates DHP and THF, respectively, together with ATP and glutamate. From progress curves with respect to both enzyme concentration and time, a standard reaction for both activities containing 0.72 μM protein incubated for 60 min was adopted. This permitted accumulation of maximal counts for accurate quantitation while staying well within the period of linearity (at least 90 min; data not shown). When DHP was used as substrate, no products with mass greater than that of DHF (which, because of its lability, was seen primarily as its oxidised product folic acid in the MALDI-ToF mass spectrometer, theoretical MH^+^ = 442.4) were detectable (Fig. 1c), demonstrating that under these reaction conditions the DHF produced by the DHFS activity is not subject to further glutamylation by the FPGS activity.

### 3.2 Kinetics of the DHFS reaction; DHP substrate inhibition and dependence on co-substrate concentrations

**Fig. 2:**
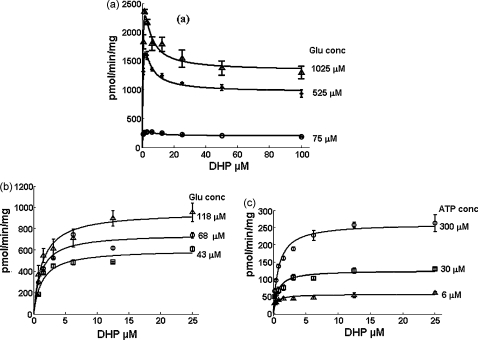
Dependence of PfDHFS activity on DHP, ATP and glutamate with DHP taken as the variable substrate. (a) ATP is kept at a constant high level of 0.5 mM in all the assays in this panel, with glutamate concentration (Glu) set at three different levels ≥75 μM. (b and c) As for (a), except ATP is kept at a constant low level of 25 μM in (b) and glutamate at a constant low level of 25 μM in (c), with stepped increments of glutamate and ATP, respectively.

Standard kinetic experiments in which two of the three substrates were present in excess, while the concentration of the third was varied, revealed that the DHFS reaction did not follow classical Michaelis–Menten kinetics. As seen in Fig. 2a, velocity *versus* [S] curves where ATP was fixed at a high concentration (0.5 mM) and glutamate was also raised to ∼1 mM, displayed characteristic biphasic kinetics, with fast initial rates that diminished as the concentration of DHP was increased. The activity peaked at about 1–2 μM DHP. Similar-shaped curves indicative of substrate inhibition were obtained when glutamate was fixed at concentrations >50 μM and ATP was varied, along with DHP (data not shown). However, neither of these co-substrates showed similar inhibition curves at higher concentrations when taken as the independent variable. Thus, this phenomenon is restricted to increases in DHP concentration only. Control experiments established that the observed kinetics were independent of the order of addition of enzyme and substrates to the reaction mix prior to the incubation period.

In order to understand the mechanism of the DHFS reaction, the activity data sets were fitted globally with a series of non-linear regression models, starting from the assumption that the sequence in which the substrates bind is random, rather than ordered, to form a quaternary enzyme–substrate complex (Supplementary Fig. 1), an assumption that is confirmed below. Initially, substrate inhibition with a second DHP molecule competitively binding to the enzyme was considered. However, though the modified formula (Supplementary Fig. 2, equation *e*) did follow the data trend, unlike the Michaelis–Menten equation (Supplementary Fig. 2, equation *f*), the fit was only improved slightly, mainly at very low DHP concentrations (compare Supplementary Fig. 3e and f). The experimental data showed that, although the rate of the reaction decreased with increasing DHP concentration, the inhibition was not complete, even at high levels of DHP (≥100 μM). Therefore, a partial inhibition term, V2 (Supplementary Fig. 2, equation *d*), was considered more appropriate. This assumes that a second binding site exists on the enzyme complex. Binding of a second molecule of DHP would change either the affinity of the enzyme complex to the normally bound substrate or the *V*_max_ of the enzyme. Introduction of this term considerably improved the fit, especially at higher DHP concentrations (Supplementary Fig. 3d). However, all of these curves, which were derived from the above equations without considering the second and third substrates (ATP and glutamate), gave at best only imperfect fits to the data sets, suggesting that the binding of the different substrates may also exert allosteric effects on the enzyme. Of the equations that took this into account (Supplementary Fig. 2, equations *a*, *b* and *c*, graphed in Supplementary Fig. 3), partial substrate inhibition in the presence of all three substrates, as in equation *a*, gave an extremely good fit with an *R*^2^ value of 0.95 (Supplementary Fig. 3a). The next best fit (equation *b*), where fully competitive substrate inhibition in the presence of three substrates was modelled, gave a much poorer *R*^2^ value of ∼0.6 (Supplementary Fig. 3b). Over a broad range of conditions where concentrations of ATP from 6 μM to 5 mM and of glutamate from 25 μM to 1 mM were tested in various combinations, the best-fit model, *a*, gave a dissociation constant, *K*_A_, for DHP of 0.50 ± 0.06 μM for the substrate binding, and a dissociation constant, *K*_ABCA_, of 1.25 ± 0.37 μM for the substrate (DHP) inhibition (*n* = 15 with *R*^2^ > 0.90 in each case). Due to the substrate inhibition feature of the system, there is no conventional substrate *K*_m_ term in this equation.

That the extent of the DHP substrate inhibition phenomenon seen above is dependent on ATP and glutamate concentrations is also clearly seen in Fig. 2b and c. When either ATP or glutamate concentrations were lowered to ≤25 μM, as in (b) and (c), respectively, such inhibition by DHP was no longer significant. Data obtained under these conditions were found to fit well to the conventional Michaelis–Menten equation, enabling the calculation of apparent *K*_m_ values for the three substrates, because here the reaction was found to be first order with respect to DHP concentration. Using a non-linear fit to the classical Michaelis–Menten equation, we determined an apparent *K*_m_ for DHP under these conditions to be 0.88 ± 0.26 μM, quite closely comparable to the dissociation constants calculated above for DHP binding at higher ATP/glutamate concentrations in the partial substrate inhibition model. The apparent *K*_m_ values for ATP and glutamate were 22.8 ± 2.7 μM and 5.97 ± 0.50 μM, respectively.

### 3.3 Kinetics of the DHFS reaction; substrate binding to DHFS

To study the binding of the three substrates to DHFS, this activity was further investigated in a series of assays where each of the three substrates in turn was taken as the variable substrate (*x*-axis), maintaining the concentration of one of the other two constant and increasing the third in steps to yield double-reciprocal plots that are indicative of the enzyme mechanism. These experiments were done under the above-mentioned conditions of low substrate concentrations, where the reaction data fit to the Michaelis–Menten equation. All combinations of conditions gave rise to convergent plots rather than parallel lines, as exemplified in Supplementary Fig. 4. These data confirm that the binding of the substrates DHP, ATP and glutamate occurs sequentially as depicted in Supplementary Fig. 1, giving rise to the quaternary complex anticipated in the modelling exercise, and exclude the possibility of a ping-pong mechanism.

### 3.4 Kinetics of the FPGS reaction and competition between the substrates of DHFS and FPGS

**Fig. 3:**
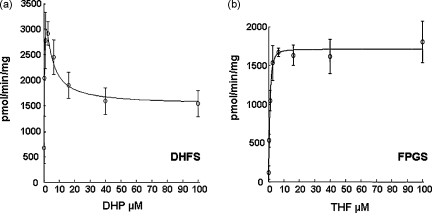
Comparison of DHFS and FPGS activities under identical conditions of high co-substrate concentrations. ATP is at 5 mM, glutamate at 275 μM. The data are fitted to the three-substrate, substrate partial inhibition model of Supplementary Fig. 2, equation *a*.

Similar experiments to the above were conducted using THF as substrate to assay the FPGS activity of PfDHFS–FPGS. Unlike the case for the DHFS activity, no unusual kinetics were observed in parallel assays on the same enzyme preparation, either under low or high substrate concentrations (Fig. 3). Velocity *versus* [S] curves fitted well to the Michaelis–Menten equation, yielding an apparent *K*_m_ value of 0.96 ± 0.12 μM for THF, very similar to the *K*_A_ and apparent *K*_m_ values for DHP in the DHFS reactions above. This lack of substrate inhibition of the FPGS activity of PfDHFS–FPGS is in contrast to the marked substrate inhibition noted previously in studies with human FPGS [43,44].

**Fig. 4:**
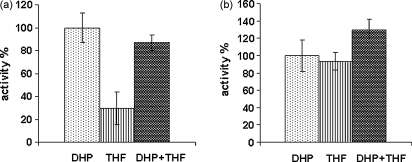
Effect of having both DHFS and FPGS substrates together in the same reaction compared to when present alone. From left to right in (a) 10 μM DHP, 10 μM THF, 10 μM DHP + 10 μM THF; (b) 100 μM DHP, 100 μM THF, 100 μM DHP + 100 μM THF. Glutamylation activity is represented as a percentage of that seen with DHP as substrate in each case. In all cases 5 mM ATP and 275 μM glutamate were present.

Given the close similarity in the reactions catalysed by DHFS and FPGS, the similarity in the affinities of their substrates and data from studies of bacterial DHFS–FPGS proteins suggesting that the catalytic sites of these activities are at the very least closely linked [21,24], we measured the rate of glutamylation in the presence of equimolar amounts of both DHP and THF substrates at two concentrations differing by an order of magnitude (Fig. 4a and b). In both cases, no significant additive effect was seen in the rate of glutamate incorporation measured relative to those seen for the same concentration of each substrate alone, consistent with there being only a single site of glutamylation available for either substrate at any given time.

### 3.5 Substrate specificity of the DHFS–FPGS activities

**Fig. 5:**
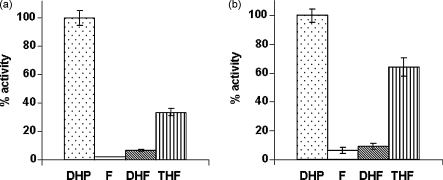
Substrate specificity of PfDHFS–FPGS. Glutamylation of equal concentrations of DHP, folic acid (F), DHF and THF by PfDHFS–FPGS under identical conditions: (a) 25 μM DHP, F, DHF or THF, 25 μM ATP and 18 μM glutamate; (b) 100 μM DHP, F, DHF or THF, 2 mM ATP and 200 μM glutamate. Glutamylation activity is represented as a percentage of that seen with DHP as substrate in each case.

To establish the specificity of PfDHFS–FPGS, equal concentrations of substrates varying in the oxidation level of the pterin moiety were compared, i.e. DHP, DHF, THF and folic acid. The activities were studied under both low and high concentration conditions of the test substrates and of the co-substrates ATP and glutamate (Fig. 5a and b). Under both of these conditions, the highest level of glutamylation activity was seen on DHP, the natural substrate of DHFS, followed by THF, the natural substrate of FPGS. In contrast, both folic acid and DHF were very poor substrates for the polyglutamylation activity of the latter. Especially under low substrate conditions, incorporation of glutamate onto folic acid was insignificant. Similarly, although DHF, the product of the DHFS activity, is in the active centre after the DHFS reaction, it seemingly cannot be efficiently polyglutamylated by the FPGS activity. Thus, using the same concentrations of DHF and THF as substrates for FPGS, the enzyme produced only 21% under low substrate concentrations (25 μM of either) and 12% under high substrate concentrations (100 μM of either) of polyglutamylated forms of folate from DHF compared with that produced from THF. Although some incorporation onto DHF does occur in the above experiments, the concentrations used are artificially high in both cases—about two orders of magnitude higher than the concentration of DHF that could be produced in our assays from the same concentration of DHP via DHFS over a 1 h incubation period. This is consistent with the MALDI-ToF mass spectrometry analysis of the reaction products (Fig. 1c), where no trace of polyglutamylated products was seen when DHP was used as substrate under these conditions. These experiments confirm that the reduction status of the folate moiety is critical for the polyglutamylation reaction, with the fully reduced tetrahydrofolate much the preferred form for the plasmodial FPGS activity. This is in marked contrast to the properties of human FPGS, which is able to polyglutamylate the dihydro-form of folic acid with efficiencies comparable to that for the tetrahydrofolate form, and can also modify the fully oxidised form to a significant, albeit lesser, degree [36,42].

### 3.6 Methotrexate as a possible substrate for PfFPGS

**Fig. 6:**
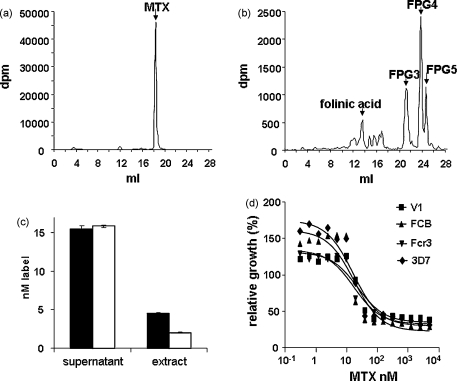
MTX accumulation in, and inhibition of, *P. falciparum*. A predominantly ring-stage culture of FCB isolate was divided equally and each half grown for 20 h in the presence of (a) ^3^H-MTX or (b) ^3^H-folinic acid in parallel and extracts analysed by HPLC. The arrows indicate the position of MTX and the mono- and polyglutamylated forms (FPG3-5) of folinic acid. (c) Accumulation of MTX (black) in parasite cells compared with folinic acid (white). Counts in the culture supernatant (converted to nM) indicate the (equal) initial label concentrations to which each culture was exposed; ‘extract’ represents the counts extracted from the equal numbers of parasites in each pellet labelled with either MTX or folinic acid after the incubation. (d) Dose–response curves of different strains of cultured malaria parasites to increasing MTX concentration. Values are relative to growth in the absence of drug, taken as 100%. For reasons as yet unclear, extremely low levels of MTX (around 1 nM) have a stimulatory effect on growth.

The above analysis showed that plasmodial FPGS is very poor at adding further glutamate residues to folic acid and thus differs importantly from human FPGS in this respect. This observation led us to test whether methotrexate (MTX) could act as a substrate for the plasmodial enzyme. MTX is a fully oxidised folic acid analogue extensively used in anti-cancer antifolate therapy, whose efficacy depends not only on its binding to the DHFR of rapidly dividing cancer cells, but also on its polyglutamylated forms binding to other enzymes in the folate pathway [45]. The FPGS in human cells can convert MTX into such forms, which, as in the case of folates themselves, are more efficiently retained in the cell and also show markedly increased affinity to certain folate-dependent enzymes [46], such as thymidylate synthase [47], 5-amino-4-imidazolecarboxamide ribotide transformylase (AICAR formyltransferase), involved in purine biosynthesis [48], or the enzymes of methionine biosynthesis [12]. The relevance to malaria is that the use of MTX has been advocated as a cheap antimalarial that could be safely used in doses far lower than necessary in cancer chemotherapy, and it has thus been envisaged that the parasite could also convert MTX into polyglutamylated forms and therefore enhance its efficacy by broadening its range of targets, as in the case of cancer cells [49]. We therefore tested MTX in two ways. First, using radiolabelled MTX in parasite cultures in parallel with a near-equimolar concentration of radiolabelled folinic acid (5-formylTHF), we found by HPLC analysis that no polyglutamylated versions of MTX could be detected after overnight incubation (Fig. 6a). In contrast, cultures supplemented with ^3^H-folinic acid generated a large amount of labelled polyglutamylated folates up to the pentaglutamate level (Fig. 6b), as seen in previous reports [8,9,38]. Indeed, the conversion of ^3^H-folinic acid to such forms over this period was almost complete. Moreover, when the efficiency of uptake and retention of the two radiolabelled compounds was compared, MTX was found to have accumulated in the parasites to somewhat higher levels than folinic acid (Fig. 6c), showing that it is not the transport into the cell that limits the polyglutamylation of MTX, but the lack of subsequent processing. This result clearly indicated that MTX is not a substrate for the plasmodial FPGS activity.

To further investigate any possible interaction between MTX and PfDHFS–FPGS, MTX was tested as a potential inhibitor in individual DHFS and FPGS assays. However, neither the DHFS nor FPGS activities showed any sensitivity to this drug, even at concentrations as high as 1.0 mM (Supplementary Fig. 5a and b). In contrast, growth of *P. falciparum* cultures was highly sensitive to MTX, with average IC_50_ values of ∼40 nM for four different strains of the parasite (Fig. 6d), as seen previously with other strains [50], i.e. about 25,000 times lower, presumably as a result of its known, very tight binding to DHFR (*K*_i_ < 1 nM), regardless of whether this enzyme carries the mutations that confer PYR resistance [51]. This indicates a complete absence of either allosteric or competitive effects of MTX on the parasite DHFS–FPGS enzyme. We conclude that if MTX has more than one target in *P. falciparum*, as has been suggested, it is not mediated via polyglutamylated forms of the drug, unlike in mammalian systems, and further that the DHFS and FPGS activities are unaffected by this antifolate agent.

### 3.7 Inhibition of the PfDHFS–FPGS activities by two novel folate analogues

**Fig. 7:**
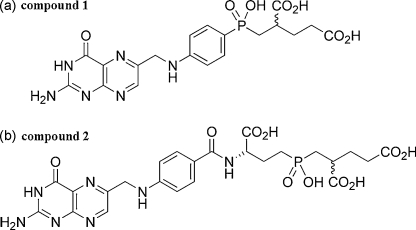
Structures of the putative PfDHFS–FPGS inhibitors, compounds **1** and **2**. These are folate analogues incorporating aryl- and alkylphosphinic acid moieties [**1** and **2**, respectively] to simulate postulated unstable tetrahedral intermediates for the two activities [31,33]. They are shown here in their oxidised forms; they were reduced to their 7,8-dihydropterin forms for the inhibition studies.

**Fig. 8:**
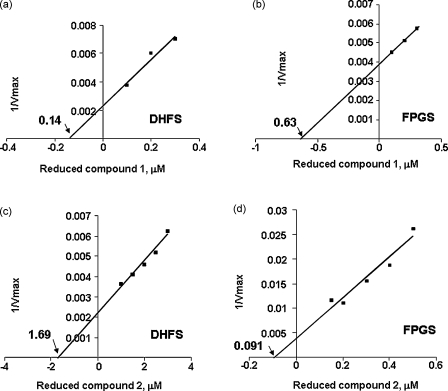
Secondary plots of 1/*V*_max_ of PfDHFS and PfFPGS activities against varying concentrations of **1** and **2** for the determination of *K*_i_ values of the inhibitors. In order to obtain *K*_m_ and *V*_max_ data (see Supplementary Fig. 7), DHP was used as the variable substrate in (a) and (c) for DHFS activity; THF in (b) and (d) for FPGS activity. ATP = 50 μM, glutamate = 25 μM. H_2_-**1** has a *K*_i_ of 0.14 μM for DHFS and a *K*_i_ of 0.63 μM for FPGS activity. H_2_-**2** has a *K*_i_ of 1.69 μM for DHFS activity and a *K*_i_ of 0.091 μM for FPGS activity, as indicated by arrows.

To date, the reactions catalysed by PfDHFS–FPGS have not been exploited, nor even explored, as possible targets for antimalarial inhibitors. Given that DHFS has no counterpart in mammals and that the parasite FPGS activity shows properties significantly different from those of human FPGS, identifying effective and selective inhibitors of these activities would be an attractive goal. We therefore investigated two novel compounds that had been designed as folate analogue inhibitors by mimicking the unstable tetrahedral intermediates derived from the acyl phosphate intermediates formed during DHFS- and FPGS-catalysed ligation of glutamic acid to DHP and THF, respectively [52]. Thus, two tetrahedral phosphinic acids (**1** [31] and **2** [33]; Fig. 7) were evaluated as predicted inhibitors of PfDHFS (**1**) and PfFPGS (**2**), respectively. In exploratory experiments, the 7,8-dihydro derivative of **1** exhibited an IC_50_ value of 0.41 μM for the DHFS activity of the recombinant protein while the value for the 7,8-dihydro derivative of **2** against the FPGS activity was 0.39 μM (Supplementary Fig. 6). The specificity of these two inhibitors was more rigorously studied by using *V*_max_ values in secondary plots to calculate the *K*_i_ values for both compounds against both activities. The data showed that reduced **1** was about 5-fold more potent in inhibiting the DHFS activity than the FPGS activity. Thus, it had a *K*_i_ value of 0.14 μM for the former and one of 0.63 μM for the latter. Conversely, reduced **2** was a much more potent inhibitor of FPGS activity (almost 20-fold), with a *K*_i_ value of 0.091 μM for the FPGS activity and of 1.69 μM for the DHFS activity (Fig. 8). These figures were derived under low substrate concentration conditions where data fit to the Michaelis–Menten equation was good (see above), but were little changed at high concentrations of ATP and glutamate (data not shown), where *V*_max_ values were determined using the full equation of model *a* in Supplementary Fig. 2.

To assess the mode of binding of the two drugs to PfDHFS–FPGS, either DHP was used as the variable substrate for the DHFS activity or THF as that for the FPGS activity with the inhibitors used in a series of constant concentration steps in each data set to yield the double-reciprocal plots of Supplementary Fig. 7. Despite converging to points in all of the plots, these lie off both the *x*- and *y*-axes, as both inhibitors induced changes in both the *K*_m_ and the *V*_max_ values of the reaction. This indicates that the effects of the drugs are not uncompetitive, but neither are they fully competitive nor fully non-competitive, with both apparently exerting their effect through a mixed mode of competitive action on the bifunctional DHFS–FPGS enzyme of the parasite.

## 4 Discussion

Folate biosynthesis and metabolism is a long-established clinical target for antimalarial intervention. We have demonstrated previously that the malarial genome encodes a bifunctional DHFS–FPGS as a component of its folate pathway and verified its predicted roles by complementation experiments in mutants of yeast and *E. coli* defective in these activities [30,34]. However, a thorough kinetic study has not yet been carried out on this protein, whose functions are critical to both folate synthesis and folate modification, from this or any other protozoan parasite. We first studied the binding of the substrates of the PfDHFS activity by systematically varying the concentrations of the three compounds involved, DHP, ATP and glutamate. Our data are consistent with their binding sequentially to form a quaternary complex, with DHP having the highest affinity among the three substrates. This would be consistent with a low level of synthesis of DHP in the parasites relative to the availability of ATP and glutamate. However, we demonstrate that the plasmodial DHFS activity is an unusual enzyme type in that accurate modelling of its kinetics required the concept of a second DHP binding site to be introduced together with the assumption that the binding of this second site by DHP inhibits the enzyme's activity, but that such inhibition is only partial, even at high DHP concentrations. A similar phenomenon has been observed for DHPS from *Staphylococcus aureus* with respect to pAB [53] and *Cryptosporidium parvum* inosine monophosphate dehydrogenase with respect to NAD [54]. In our experiments, substrate inhibition became significant at DHP concentrations higher than ∼2 μM and was most pronounced when the co-substrates ATP and glutamate were readily available at the higher concentrations expected *in vivo*. Thus in general, ATP concentrations are in the mM range in most cell types investigated [55], and this is also true of *P. falciparum* blood stages, where an average level of ∼2.5 mM has been measured for parasites cultured in physiological glucose [56]. Similarly, studies on *P. lophurae* indicate that glutamate levels are normally at least 1 mM and probably higher in the cytosol [57]. The unusual kinetics we observe suggest that under normal *in vivo* conditions, production of the folate moiety might be a step in the biosynthetic pathway at which a certain degree of negative feedback is operational, depending upon the (unknown) concentrations of DHP. In contrast, no evidence was seen for substrate inhibition in the FPGS reaction with THF as substrate under any conditions, quite different to what is observed with human FPGS [43,44,58]. The apparent *K*_m_ values of ∼1 μM that we determined for the parasite DHFS and FPGS activities with respect to their principal substrates are comparable to those seen with other homologues, e.g. ∼0.4–6 μM for DHP with bacterial DHFS [22,24,37] and ∼2–5 μM for THF with bacterial and mammalian FPGS [37,42,59,60]. Values of *V*_max_ were very variable, depending upon the enzyme preparation and its age, reflecting significant instability, as also reported for a range of other DHFS/FPGS molecules, both prokaryotic and eukaryotic [22,25,28,36,59]. This, together with the DHP inhibition phenomenon observed here for the PfDHFS activity made meaningful comparisons with values reported for other systems difficult. However, the highest turnover numbers we calculated were 0.04 s^−1^ (uninhibited) and 0.01 s^−1^ (substrate inhibited), significantly slower than reported for human and bacterial enzymes (0.6–0.8 s^−1^ [25,37,42]), but similar to figures of 0.02–0.03 s^−1^ reported for the *P. falciparum* HPPK–DHPS bifunctional enzyme that precedes DHFS in the folate biosynthetic pathway [61,62]. Another enzyme in the thymidylate cycle component of the *P. falciparum* folate pathway, serine hydroxymethyltransferase (SHMT), exhibits faster turnover numbers (0.16–0.74 s^−1^ [63,64]), but are similarly calculated as being, on average, about 30 times slower than their human or bacterial equivalents [63].

Our studies of substrate specificity also indicate that the product of the DHFS step, DHF, despite its location at the catalytic site of the DHFS activity, almost certainly must dissociate and undergo reduction by DHFR to THF, before rebinding in this form to the site of FPGS activity for conversion into polyglutamylated forms. Whether the two active sites are discrete or overlapping in bacteria with a bifunctional DHFS–FPGS has been the subject of some controversy [21,23,24], but our data on the parasite protein (Fig. 4) are more consistent with the latter view, that DHP and THF must occupy the same or extensively overlapping catalytic sites. This in turn raises the question as to how the relative levels of folate production and subsequent polyglutamylation are controlled, if at all, in this organism.

The most obvious difference between parasite and host with regard to these aspects of folate metabolism is that, as in the case of *E. coli* and other bacteria, the plasmodial protein has two functions whereas the human enzyme has only the FPGS activity and entirely lacks a DHFS, along with other enzymes of *de novo* folate synthesis. However, there is a significant level of similarity between human FPGS, DHFS–FPGS from the malarial parasite and DHFS or FPGS enzymes from other organisms in their primary protein sequences [34]. They all have an active site located in the same area of the enzyme and the binding of ATP and glutamate is conserved among all of them. Importantly though, the substrate specificity studies reported here demonstrate that, in addition, the parasite FPGS has properties that differ significantly from those reported for the orthologue of its host, particularly with respect to the oxidation state of the folate to be glutamylated, further justifying the bifunctional parasite molecule as a putative novel drug target. Aryl phosphinic acid analogues of folic acid and several antifolates have been synthesised as potential inhibitors of DHFS [31]. With targeting of bifunctional DHFS–FPGS in mind, including that of *P. falciparum*, we tested for the first time such inhibitors against the plasmodial target activities. Thus, using the aryl phosphinic analogue of folic acid (**1**), we demonstrated that this compound in its 7,8-dihydro reduced form (but not its oxidised form) strongly inhibited the DHFS activity of the parasite with a *K*_i_ of 140 nM, but was about 5-fold less effective against the FPGS activity. We also tested an alkyl phosphinic acid analogue of pteroylglutamyl-γ-glutamate (**2**), an inhibitor originally designed to target the human FPGS activity [32,33,65] against both of the activities of PfDHFS–FPGS. Consistent with its design, this analogue, again in its reduced form, showed a marked preference for the plasmodial FPGS activity with a *K*_i_ of 91 nM, almost 20-fold more potent than that measured for the DHFS activity (1.69 μM). Given how poor we found DHF itself to be as a substrate for PfFPGS, the striking effectiveness of the dihydro-form of **2** against this activity is perhaps surprising, and suggests that in this case the nature of the heterocycle is less important for binding than is the phosphinate mimic of the tetrahedral intermediate. These *in vitro* inhibitor studies on purified enzyme serve as an important proof of principle, but in preliminary experiments, the reduced compounds were unable to significantly inhibit parasite growth in culture, probably due to instability over long periods at 37 °C and/or insufficient cell permeability. Such analogues would therefore require further appropriate structural modifications to overcome these current limitations.

The study using MTX as a candidate inhibitor yielded several interesting results. This major anti-cancer antifolate is reported to inhibit multiple enzymes in mammalian cells in addition to its traditional target of DHFR [18]. This secondary mechanism of MTX inhibition is due to its polyglutamylation by cellular FPGS activity, increasing its affinity for other key molecules, including TS and other folate-dependent enzymes [46]. Moreover, in cells that can potentially develop resistance to MTX by pumping the unaltered drug outside of the cell membrane, polyglutamylation enhances its accumulation by virtue of the increases in negative charge. Our study of the ability of plasmodial FPGS to catalyse MTX polyglutamylation, both *in vitro* and *in vivo*, again highlighted major differences between parasite and human FPGS activities. No polyglutamylated forms of MTX could be detected after overnight cultivation with the ^3^H-MTX label whereas the conversion of a near-equimolar concentration of ^3^H-folinic acid, a folate with a fully reduced (tetrahydro-) pterin ring, was almost complete under identical conditions, a phenomenon that we showed was not due to poor transport and accumulation of the drug in the parasite, which actually exceeded that of folinic acid. In fact, we could find no evidence for any interaction between PfDHFS–FPGS and MTX, nor more than a very low level of activity on folic acid, which MTX resembles closely, especially in the fully oxidised nature of the pterin ring. Whether MTX inhibits any enzyme in the parasite folate pathway other than its classical target DHFR, or indeed any other non-folate related activities, remains to be established, but we can confidently exclude MTX inhibition of PfDHFS–FPGS and any role of polyglutamylated forms of this drug in its powerful antimalarial effect.

Taking the above studies on phosphinic acid folate analogues and MTX together, our results emphasise that, despite the close similarities in their function and the likely extensive overlap in their catalytic site, the DHFS and FPGS activities of the parasite can be differentiated from each other at the inhibitor level. Moreover, the kinetic and substrate specificity experiments reveal that the parasite FPGS shows significant differences relative to its human orthologue. These observations, together with the absence of DHFS activity in humans, open up the possibility of developing inhibitors against the bifunctional DHFS–FPGS protein that are highly specific for the parasite.
