# Supplementary material for: Characterisation of the bifunctional dihydrofolate synthase–folylpolyglutamate synthase from Plasmodium falciparum; a potential novel target for antimalarial antifolate inhibition
Source: Mol Biochem Parasitol. 2010 Jul;172(1):41–51. doi: 10.1016/j.molbiopara.2010.03.012 (PMC2877875; doi:10.1016/j.molbiopara.2010.03.012)
Supplement: Supplementary file 1 [file mmc1.pdf]

SUPPLEMENTARY DATA for:

Characterisation of the bifunctional dihydrofolate synthase-folylpolyglutamate synthase from *Plasmodium falciparum*; a potential novel target for antimalarial antifolate inhibition

Ping Wang[a], Qi Wang[a], Yonghong Yang[b,†], James K. Coward[b], Alexis Nzila[c], Paul F. G. Sims[a] and John E. Hyde[a,*]

[a]Manchester Interdisciplinary Biocentre, Faculty of Life Sciences, University of Manchester, 131 Princess Street, Manchester M1 7DN, UK.

[b]Departments of Medicinal Chemistry and Chemistry, University of Michigan, 930 N. University, Ann Arbor, MI 48109-1055 USA.

[c]KEMRI, Wellcome Trust Collaborative Research Programme, Kilifi 80108, Kenya.

[†]Current address: Carestream Health Inc., 4 Research Dr., Woodbridge, CT 06525, USA.

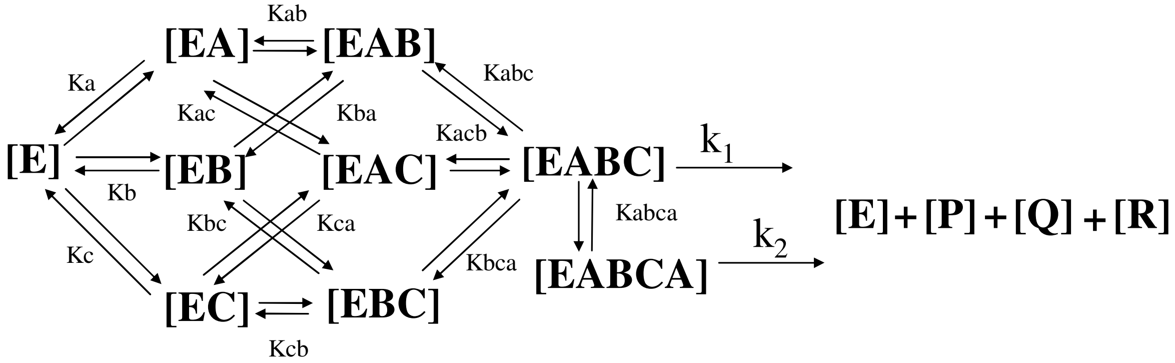

Supplementary Fig. 1

PfDHFS enzyme reactions. A: DHP; B: ATP; C: glutamate; P:DHF; Q:ADP; R: Phosphate; E: DHFS. All the K symbols represent equilibrium constants, k_1 is the catalytic rate constant for uninhibited DHFS, k_2 is the catalytic rate constant for substrate-inhibited DHFS.

a.
```
y = ((Vmax+(a/Kabca)*V2)*a*b*c)/(Ka*Kab*Kabc+Kab*Kabc*a+Kba*Kabc*b
                                 +(Ka*Kab*Kabc/Kc)*c+Kabc*a*b+Kacb*a*c
                                 +(Kba*Kabc/Kbc)*b*c+(1+a/Kabca)*a*b*c)
```

b.
```
y = Vmax*a*b*c/(Ka*Kab*Kabc+Kab*Kabc*a+Kba*Kabc*b
               +(Ka*Kab*Kabc/Kc)*c+Kabc*a*b+Kacb*a*c
               +(Kba*Kabc/Kbc)*b*c+(1+a/Kabca)*a*b*c)
```

c.
```
y = Vmax*a*b*c/(Ka*Kab*Kabc+Kab*Kabc*a+Kba*Kabc*b
               +(Ka*Kab*Kabc/Kc)*c+Kabc*a*b+Kacb*a*c
               +(Kba*Kabc/Kbc)*b*c+a*b*c)
```

d.
```
y = (((Vmax+(V2/Kaa)*a))*a)/(Ka +(1+a/Kaa)*a)
```

e.
```
y = (Vmax*a)/(Ka+(1+a/Kaa)*a )
```

f.
```
y = (Vmax*a)/(Ka+a)
```

Supplementary Fig. 2

Formulae used to fit the data in Supplementary Fig. 3; a: DHP, b: ATP, c: glutamate. V_{max} is the maximal activity of the enzyme without substrate inhibition. V2 is the maximal activity when the enzyme is inhibited by substrate binding at the second site. All the K symbols represent the different equilibrium constants depicted in Supplementary Fig. 1.

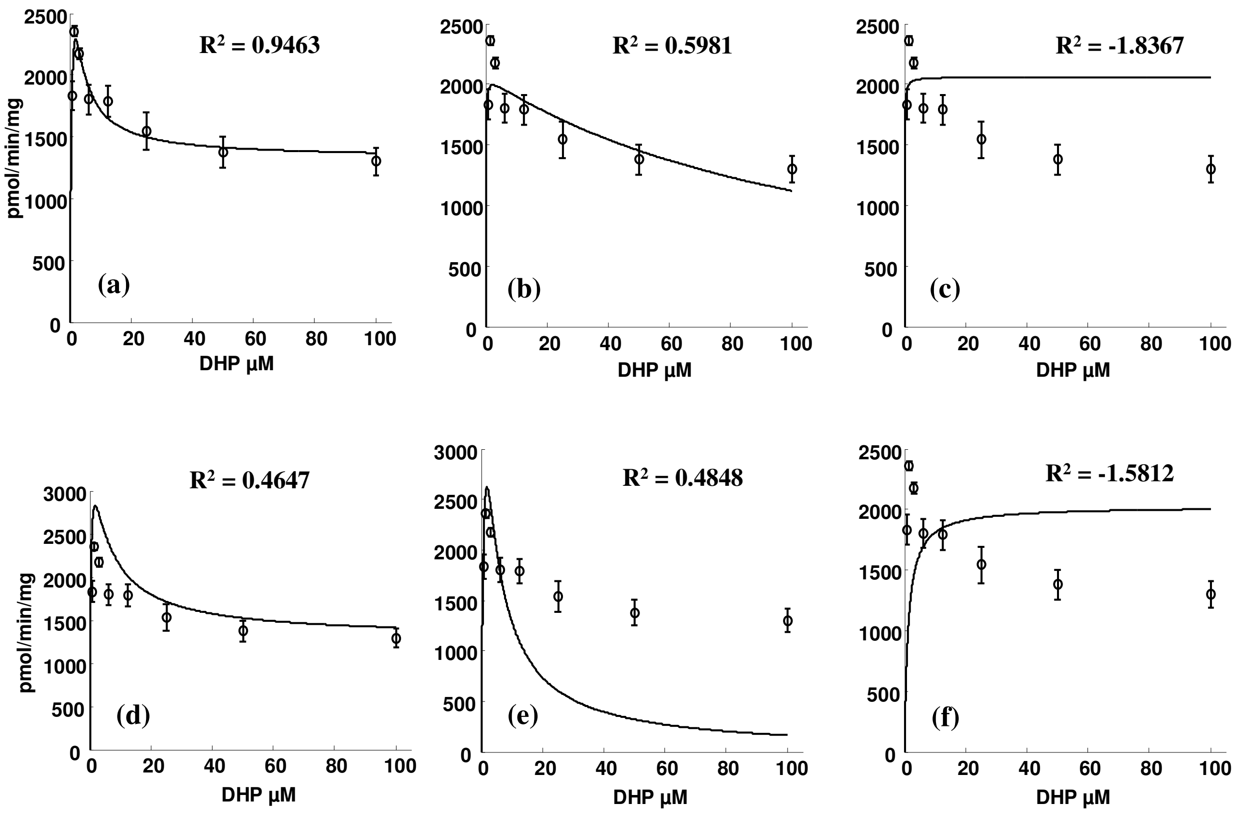

Supplementary Fig. 3

Kinetic models of Supplementary Fig. 2 fitted to PfDHFS activity data; ATP = 0,5 mM, glutamate = 1.025 mM). All six models are fitted to one set of data to compare their goodness of fit, quantified as a coefficient of determination ($R^2 = 1 - SS_{reg}/SS_{tot}$, as used, e.g., in GraphPad™) shown for each case.

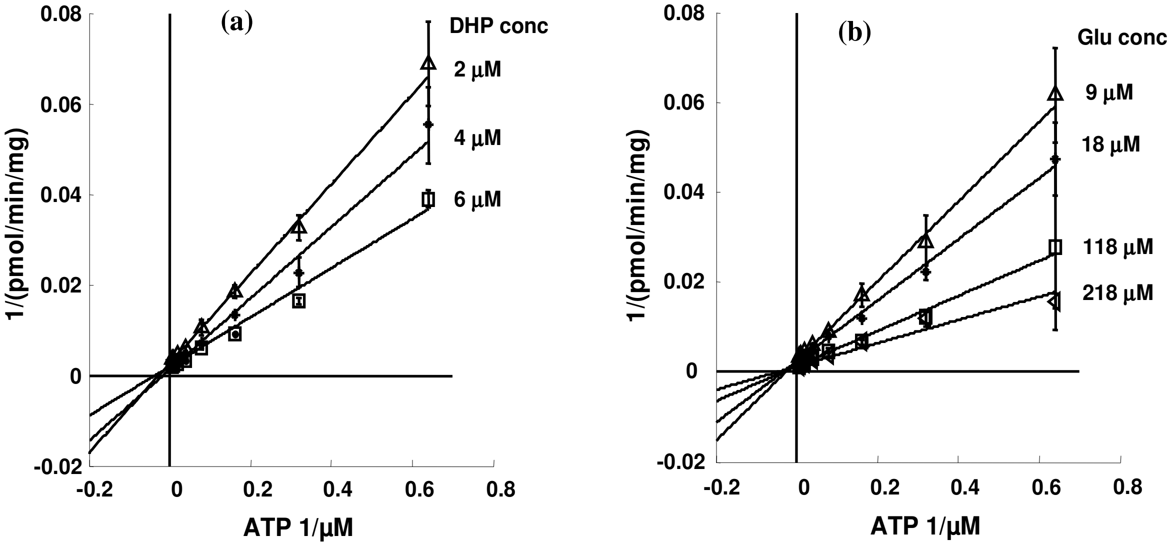

Supplementary Fig. 4

Substrate binding study of PfDHFS carried out when one substrate is taken as the independent variable and one of the others is increased in steps as indicated. The third (fixed) substrate concentration in (a) is 18 μM glutamate and in (b) 10 μM DHP.

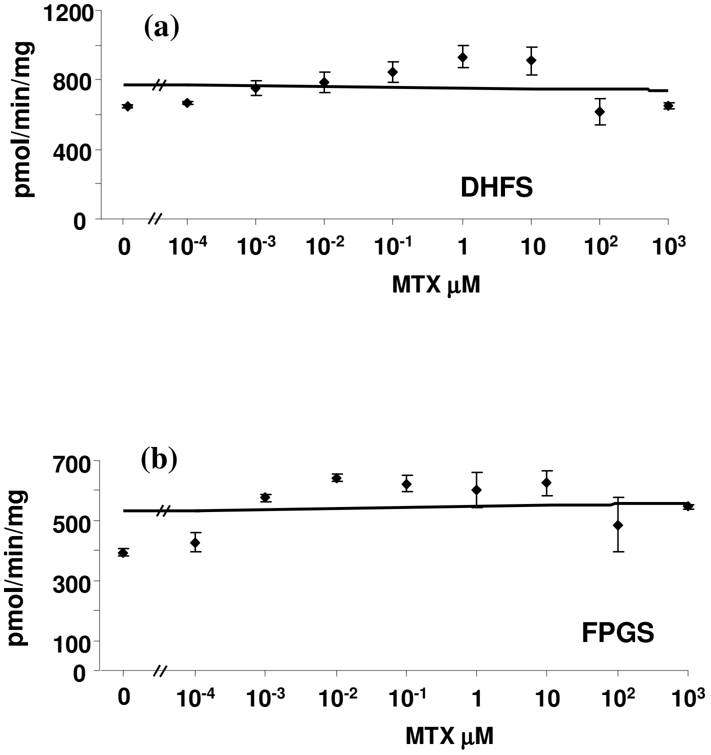

Supplementary Fig. 5

Effect of MTX on DHFS and FPGS activities, respectively; DHP (a) or THF (b) was set at 10 μM, ATP at 5 mM and glutamate at 275 μM. Lines of best fit are plotted.

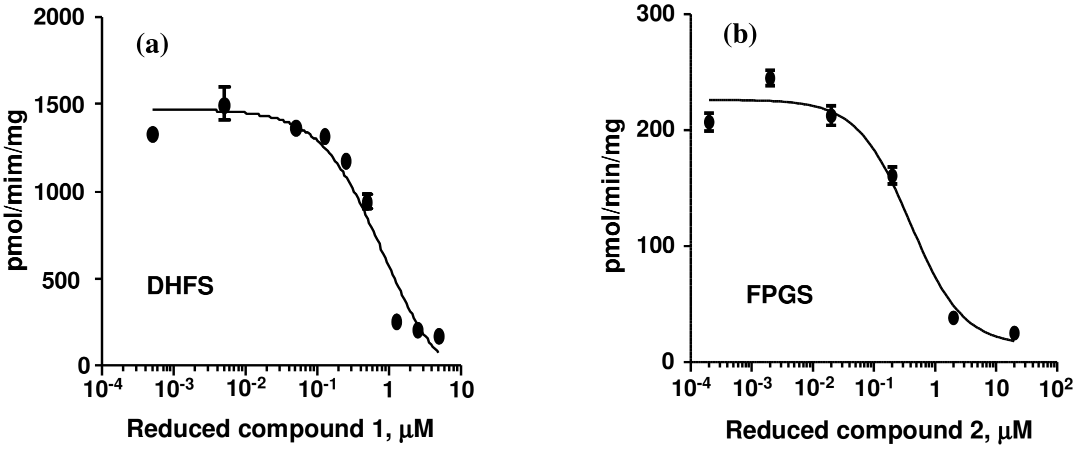

Supplementary Fig. 6

Inhibition of (a) PfDHFS activity with the dihydro- form of **1** and (b) PfFPGS activity with the dihydro- form of **2**. DHP and THF were at 50 µM respectively, with 50 µM ATP and 25 µM glutamate. These curves yielded IC_{50} values for **1** of 0.41 µM and for **2** of 0.39 µM.

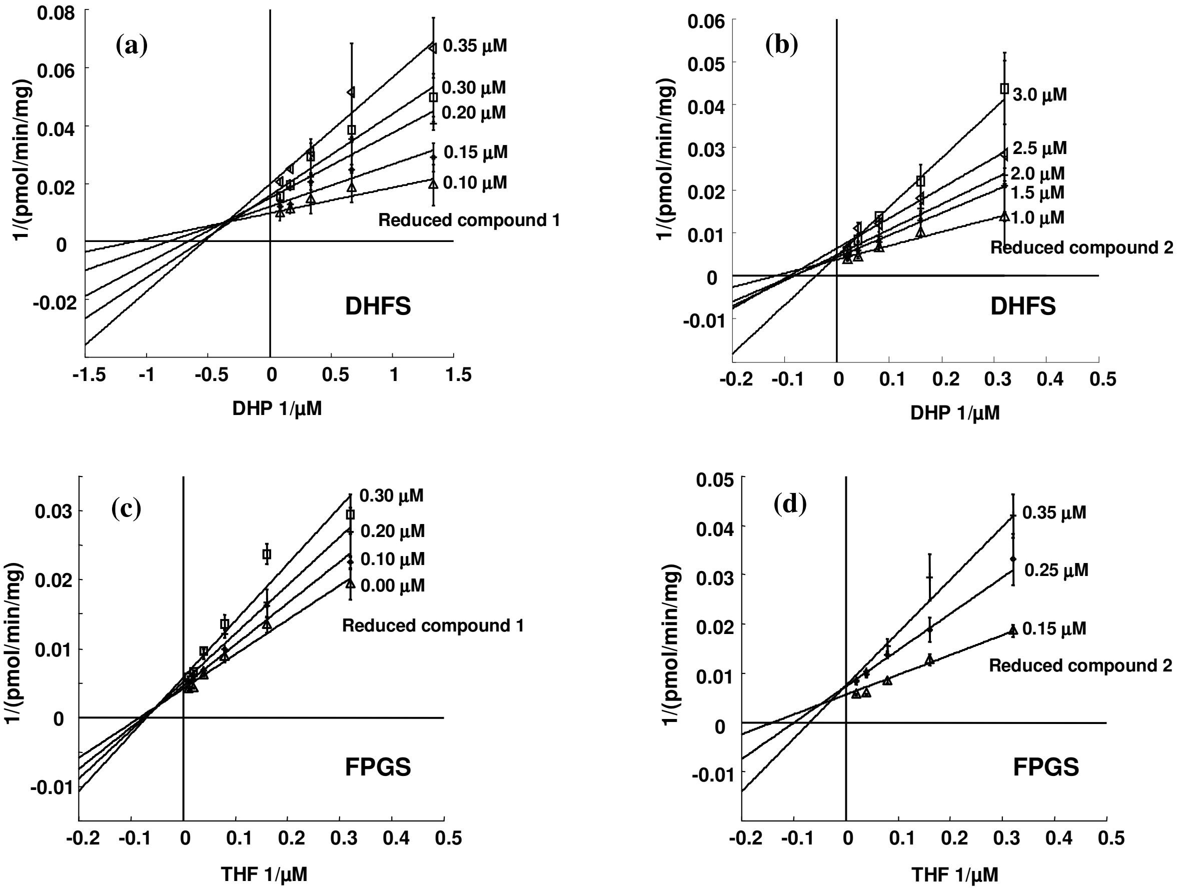

Supplementary Fig. 7

Inhibitor binding study of reduced **1** and **2** (dihydro- forms) on the PfDHFS-FPGS enzyme. Double reciprocal plots of reaction rate *versus* substrate concentration from triplicate assays under different concentrations of inhibitor (figures to the right of each graph) were used to establish the nature of the competitive relationship between the drug and the substrate in binding to the enzyme. (a) & (b) show DHFS activity with DHP, (c) & (d) show FPGS activity with THF, each with different concentrations of H_2**1** and H_2**2** respectively. ATP = 50 µM, glutamate = 25 µM.
